# The timings of host diapause and epidemic progression mediate host genetic diversity and future epidemic size in *Daphnia–*parasite populations

**DOI:** 10.1098/rspb.2022.2139

**Published:** 2023-03-29

**Authors:** Stuart K. J. R. Auld, June Brand, Luc F. Bussière

**Affiliations:** ^1^ Biological & Environmental Sciences, University of Stirling, Stirling, UK; ^2^ Biological & Environmental Sciences & Gothenburg Global Biodiversity Centre, University of Gothenburg, Gothenburg, Sweden

**Keywords:** phenology, evolution, mesocosms, parasitism, temporal dispersal

## Abstract

Epidemics commonly exert parasite-mediated selection and cause declines in host population genetic diversity. This can lead to evolution of resistance in the long term and smaller subsequent epidemics. Alternatively, the loss of genetic diversity can increase host vulnerability to future disease spread and larger future epidemics. Matters are made more complex by the fact that a great many host organisms produce diapausing life stages in response to environmental change (often as a result of sexual reproduction; e.g. plant seeds and invertebrate resting eggs). These diapausing stages can disrupt the relationship between past epidemics, host genetic diversity and future epidemics because they allow host dispersal through time. Specifically, temporally dispersing hosts avoid infection and thus selection from contemporary parasites, and also archive genetic variation for the future. We studied 80 epidemics in 20 semi-natural populations of the temporally dispersing crustacean *Daphnia magna* and its sterilizing bacterial parasite *Pasteuria ramosa*, and half of these populations experienced a simulated environmental disturbance treatment. We found that early initiation of diapause relative to the timing of the epidemic led to greater host genetic diversity and reduced epidemic size in the subsequent year, but this was unaffected by environmental disturbance.

## 1. Introduction

Infectious disease epidemics can drive rapid host evolution, and in some cases host–parasite coevolution, with substantial consequences for the genetic diversity of host populations [1–6]. There are, however, significant unanswered questions concerning precisely how parasite-mediated selection affects genetic diversity of host populations in the short term (i.e. across individual epidemics), and how this could mediate the severity of disease outbreaks from epidemic to epidemic. Population surveys and infection experiments have taught us that, on one extreme, large epidemics and strong directional selection for increased host resistance can rapidly strip genetic variation from natural populations, ultimately bringing evolution to a standstill until the arrival of new genetic variants (e.g. [7]). At the other extreme, when virulent infection depends on the precise combination of host and parasite genotypes, the resulting fluctuating selection can drive partial selective sweeps and more modest declines in genetic diversity over short time scales (Red Queen dynamics); in that case, adaptation is largely fuelled by standing genetic variation and does not require new mutations [4,8–10] (but see also [11]).

Genetic diversity is important for multiple reasons: it is affected by previous bouts of selection and is also the currency of evolution and adaptation [12]. Genetic diversity can also alter the ecology of host–parasite interactions in ways that determine the size of future epidemics [13]. Specifically, parasite transmission is more likely to occur between hosts that are genetically similar [14–16]. Epidemic size, disease-driven host evolution, and host genetic diversity are thus inherently linked, making disease systems engines for eco-evolutionary dynamics [17]. However, the precise relationships between strength of ecological interactions and host genetic diversity are often difficult to discern.

Matters are further complicated by the fact that a great many organisms from diverse taxa undergo temporal dispersal by producing dormant stages (e.g. *Aedes* mosquitoes, *Austofundulus* fish, *Heterodera* nematodes, *Lepidium* grasses, *Desmarestia* algae, *Pseudophryne* toads and *Daphnia* waterfleas, as well as fungal and bacterial spores; see [18] for a review). By producing dormant stages (usually as a consequence of sexual reproduction), host mothers' enable their offspring to opt out of adverse current conditions such as high population densities, parasite epidemics or environmental degradation/disturbance [19–22]. Offspring emerge from dormancy later when conditions may be more favourable [23,24], releasing reserves of genetic variation that might otherwise be removed by selection acting on the non-dormant population [25,26].

**Figure 1.**
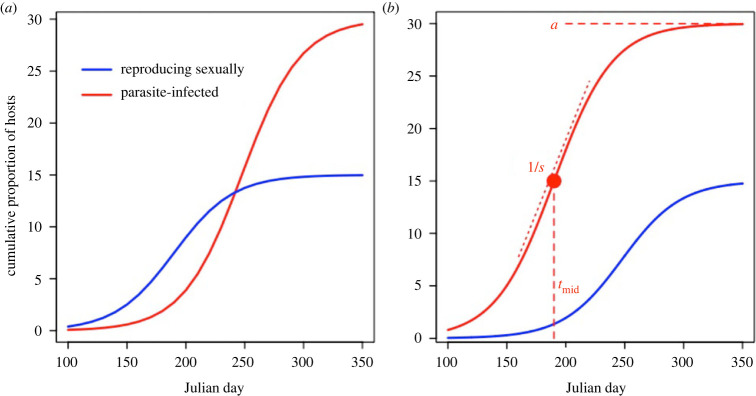
Timing of both epidemics and host sex shape parasite-mediated selection and thus the symmetry of coevolution. (*a*) the bulk of host investment in sexual reproduction occurs before epidemic onset; (*b*) the epidemic occurs before the host population invests in sex. The asymptote (*a*), midpoint (*t*_mid_) and scale (*s*) coefficients required to fit the cumulative distribution functions are also shown in panel (*b*).

The relative timing of host temporal diapause and of any parasite epidemics could thus be critical in determining the genetic diversity of future host populations. By re-supplying the future host population with archived genetic variation, temporal dispersal has the potential to dilute the effects of parasite-mediated selection. If host sex (and thus temporal dispersal) occurs early relative to epidemic progression, then the host population's overall exposure to parasite-mediated selection is reduced (figure 1*a*) and the resulting diapausing host population should harbour high genetic diversity. This is important because higher host population genetic diversity can determine the long-term persistence of populations by facilitating host evolution in the face of shifting selection [12], including selection imposed by an ever-evolving parasite population [24].

Conversely, if parasite-mediated selection occurs relatively early compared to host sex, we expect parasite-mediated selection will have a greater influence on the whole host population relative to host-mediated selection, which is little affected by host temporal dispersal (figure 1*b*). This means host genetic diversity may be reduced in both the non-dormant and diapausing fractions of the host population because more parasite-mediated selection took place before diapause could occur. The consequence is a reduction in total host diversity in the next year. It remains unclear to what extent these theoretical predictions explain natural patterns in disease systems, especially given that temporal dispersal commonly occurs as a result of sex, which itself increases host genetic diversity through recombination.

The naturally coevolving *Daphnia magna–Pasteuria ramosa* host–parasite system provides a window through which to view the effects of host temporal diapause on disease epidemics over time. *Daphnia* are freshwater crustaceans that can reproduce both sexually and asexually. *Daphnia* reproduces primarily asexually (*via* ameiotic parthenogenesis), where clonal daughters are produced throughout the season, and enter the non-dormant population immediately after being released from their mother's brood pouch. However, environmental cues such as high population density, environmental degradation/disturbance and declining photoperiod induce *Daphnia* to asexually produce males, which go on to mate with sexually receptive females [23,27,28]. Sex results in temporal dispersal, i.e. the production and release of diapausing eggs that remain dormant until favourable conditions induce hatching in subsequent years, and individual females can return to asexual reproduction after sex. *Daphnia* are also faced with strong parasite-mediated selection from coevolving parasites, such as the sterilizing bacterium *Pasteuria ramosa,* giving rise to coevolution both within individual epidemics [4,29], and across multiple years [8]. Facultative sex could therefore affect future epidemic size through two potentially overlapping mechanisms: temporal dispersal, or genetic recombination.

Here we test: (1) if environmental disturbance through physical flux affects the relative timing of host sex (diapause) and epidemic onset, and total host investment in sex; (2) if the relative timing of sex and epidemic progression or total investment in sex, i.e. the level of recombination within populations has the biggest effect on future host genetic diversity; and (3) if host population genetic diversity affects subsequent epidemic size in the following year. To achieve these aims, we used twenty semi-natural outdoor *Daphnia–Pasteuria* populations, each of which was established with an identical suite of *Daphnia* genotypes and the same initial mixed parasite population; this is part of the ongoing SODE (Stirling Outdoor Disease Experiment). We then conducted extensive and detailed monitoring of host investment in asexual and sexual (dispersing) offspring, and parasite prevalence across four consecutive seasons (a total of 80 epidemics), and genotyped representative samples of each host population early in the last three seasons.

## 2. Material and methods

### (a) Study populations

*Daphnia magna* (Straus, 1820) is a planktonic crustacean that lives in ponds and lakes throughout Europe and is host to numerous parasites [30]. We focus on the common *Daphnia* parasite, *Pasteuria ramosa* (Metchnikoff, 1888)*,* a spore-forming bacterium that sterilizes its hosts. *Pasteuria* transmission spores infect the host orally during host feeding, cross the gut epithelium [31,32] and proliferate within the haemocoel [33]. Mature *Pasteuria* transmission spores are released into the environment upon host death [34]. *Pasteuria-*infected *Daphnia* have obvious red-brown bacterial growth in their haemolymph, lack developed ovaries or offspring in their brood chamber and sometimes exhibit gigantism [33–35].

We isolated and hatched 21 diapausing *Daphnia* eggs from sediment samples collected in June 2014 (from Kaimes Farm, Leitholm, Scottish Borders, UK: 2°20′43″W, 55°42′15″N), propagated them asexually by maintaining them under favourable conditions, and genotyped them using 15 Microsatellite loci (see [36] for details). We maintained replicate lines for 12 of these genotypes in a state of clonal replication for a minimum of three generations prior to commencing the experiment, in order to minimize variation in maternal effects. These original genotypes varied in their susceptibility to *Pasteuria*, with genotypic mean proportion infected varying from 0 to 0.75 following a 48 h exposure to *Pasteuria* spores [4]. There were five replicate lines per genotype, where each replicate consisted of five *Daphnia* kept in 200 ml of artificial media [37], modified using 5% of the recommended SeO_2_ concentration; [38]. Lines were fed 5.0 ABS of *Chlorella vulgaris* algal cells per day (ABS is the optical absorbance of 650 nm white light by the *Chlorella* culture), kept at 20°C, and media was changed three times per week. The offspring from these lines were pooled according to genotype, then ten 1–3 day old animals *per* genotype were randomly allocated to an experimental pond. There were thus 120 *Daphnia* per pond (10 *Daphnia* × 12 genotypes).

The *Pasteuria* spore mix was originally established by exposing 21 Kaimes *Daphnia* genotypes their native sediment samples, collecting infected hosts, isolating transmission spores and re-exposing those spores to healthy hosts for multiple rounds of infection (See [36]). We are therefore confident the *Pasteuria* spore mix contains many strains of the bacterium.

SODE began on 2 April 2015 (Julian day 98), when the 120 × 1–3 day old *Daphnia* (10 *Daphnia* × 12 genotypes) and 1 × 10^8^ *Pasteuria* spores were added to each of 20 outdoor 1000L PVC ponds. Each pond was lined with approximately 10 cm of topsoil and filled with rainwater that had accumulated over an eight month period. On the first day of the experiment, all ponds experienced a physical flux (population mixing) treatment where mixed ponds were stirred once across the middle and once around the circumference with a 0.35 m^2^ paddle submerged halfway into the pond. Subsequently, half of the ponds were subject to the physical flux treatment once per week throughout four growing seasons (2015–2018).

We monitored a total of 80 epidemics across 4 years. Specifically, we made weekly recordings of the densities of juvenile, asexually reproducing, sexually reproducing (diapause stage producing) and *Pasteuria-*infected *Daphnia* life stages over the four growing seasons. We did this by passing a 0.048 m^2^ pond net across the diameter of the mesocosm (1.51 m) and counting the resulting *Daphnia*. If there were fewer than 100 *Daphnia* from the net sweep, we took a second sweep of the pond. *Daphnia* were made from live samples at the time of collection, and animals were returned to the ponds immediately after the count had been made. *Pasteuria-*infected *Daphnia* were diagnosed by their obvious red-brown colour, and sexually reproducing *Daphnia* were identified by the presence of a black ephippium in the brood pouch. The first season ran from 16 April to 11 November 2015; the second season ran from 15 April to 24 October 2016; the third season ran from 11 April to 11 October 2017; and the fourth season ran from 12 April to 5 October 2018. We sampled 18–25 *Daphnia* from each population on 20 May 2016, 18 May 2017 and 17 May 2018, and genotyped them using established protocols [36]; these data were used to determine the host population genetic diversity in seasons 2–4.

### (b) Analysis

All analyses were conducted using R v. 3.6.0 software [39]. First, we characterized the dynamics of epidemics and host population investment in temporal dispersal (sex) by fitting cumulative distribution functions (CDFs) to the prevalence of parasite-infected and diapause-bearing *Daphnia* in each of the 80 epidemics. Specifically, we fitted separate three-parameter logistic models to the daily cumulative proportion of *Daphnia* infected with the *Pasteuria* parasite and the daily cumulative proportion of *Daphnia* carrying diapause eggs epidemic (using the *nlme* package [40]).

$$y=\frac{a}{\left(1+e^{(t_{\mathrm{mid}}-t)/s}\right)}.$$

From each model, we extracted values for the three coefficients: the asymptote (*a*), inflection point (*t*_mid_) and scaling constant (*s*). These parameters correspond to the following biological features: the asymptote reflects overall host population investment in sex/epidemic size; the inflection point denotes the timing at which the host population is halfway through investing in sex or the mid-point of the epidemic; and the scaling constant reflects rate at which genotypes archive genetic material in the diapausing fraction of the population, and the rate at which hosts become infected (figure 1). It is important to note that coefficients of nonlinear models are not estimated independently, and often covary strongly with one another. We therefore examined the correlations among nonlinear coefficients and eliminated the synchrony parameters (see Results below) from consideration for subsequent analyses; we focused on the midpoint and asymptote parameters, as these are of most relevance to our original hypotheses.

We then studied the relationships between the properties of host sex, epidemic dynamics and genetic diversity using structural equation modelling (SEM; implemented in the *piecewiseSEM* package [41]). SEM allows the evaluation of different causal pathways between variables and therefore can quantify alternative mediating relationships that might produce similar to sociations. Our *a priori* path model was constructed using detailed knowledge of the host–parasite system in order to assess factors affecting epidemic size. The sequential nature of our dataset (with observations across four years) helped to direct the *a priori* path diagram and reduce the number of possible paths. For example, we could reasonably exclude paths where future phenomena were hypothesized to cause changes in variables measured in the past. Below we outline the variables we considered in our SEM models and their hypothesized effects.

#### (i) Physical flux

This was a basal environmental disturbance treatment that involved either within-population mixing on a weekly basis (simulating environmental disturbance) or leaving the population unstirred (unmixed control). Physical flux is known to reduce overall epidemic size and depress *Daphnia* population densities [36]. We therefore predicted that physical flux would be associated with increased host investment in sex/diapause, which is known to occur when environmental conditions degrade [42], and potentially earlier host population investment in sex/diapause; this could affect the relative timing of epidemic progression and host investment in sex (though we have no *a priori* predictions on the direction of the effect).

#### (ii) Year

Our pond populations were ecologically complex and presumably increased in complexity across years. We therefore assumed that there was considerable unmeasured variation over years that could have affected other variables of interest (particularly the relative timings of host sex and epidemic progression, total host investment in sex, future host genetic diversity and future epidemic size). We, therefore, fitted year as a simple linear effect after examining trends across years; fitting year as a factor produced no obvious qualitative differences, but it did prevent the estimation of all coefficients because of the large number of interaction terms.

#### (iii) The relative timing of temporal dispersal (sex) and epidemic

*Pasteuria* epidemics have long been known to reduce *Daphnia* population genetic diversity [36,43], and early epidemics would be most likely to castrate hosts before they had opportunity to reproduce sexually, thus having the largest impact on host genetic diversity. By contrast, early *Daphnia* population investment in sex should lead to a more genetically diverse emergent population in the following year, because sex fosters genetic recombination, and early sex would archive this genetic diversity before the clonal diversity of the non-dormant population declines due to various selection pressures [44–46] (but see also [47]). We subtracted the midpoint for sex from the midpoint for epidemic progression to derive a single index of the relative timing of these phenomena, which aligned more closely with our *a priori* hypothesis and reduced the number of paths under consideration (a real asset in light of the limited number of populations).

#### (iv) Future host diversity

We predicted that high host genetic diversity would result in smaller epidemics. Again, this prediction is built on a strong theoretical grounding [14] and existing evidence that *Daphnia* population diversity leads to smaller epidemics (though for parasites other than *Pasteuria* [16,48]).

To quantify host genetic diversity we computed the expected heterozygosity (H_exp_) on 15 microsatellite loci on samples collected from each of the 20 ponds in years 2016–2018; this was done using the *poppr* package: [49], and tested how H_exp_ varied according to year using a linear mixed effects model, where year was fitted as a fixed factor and population as a random effect. These microsatellite alleles are presumed to be selectively neutral, but might nevertheless reflect diversity at other loci under selection in our experiment, especially because of clonal propagation and predicted effects of parasite-mediated selection within years. Ponds containing high allelic diversity at microsatellite loci are likely to have retained diverse alleles at loci important to parasite resistance as well. Note that the timing of temporal diapause or epidemic progression in one year may affect epidemic size in the subsequent year, but these effects are presumed to occur at least partly *via* selection on the host population. In other words*,* our hypotheses predicted a mediating effect of genetic diversity between the relative timing of sex and epidemics, and future epidemic size (see the lack of direct path between these effects in figure 3*a*).

**Figure 2.**
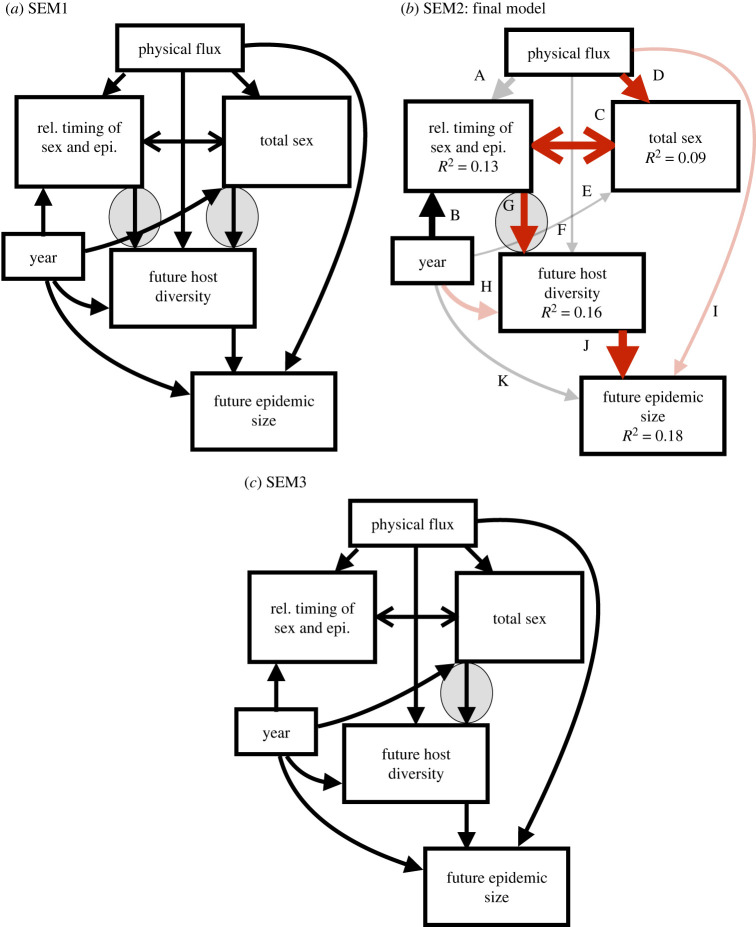
Path diagrams for: (*a*) SEM1 testing both total host sex and the relative timings of host population investment in sex and epidemic onset affect host genetic diversity and epidemic size in the following year; (*b*) SEM 2 testing the effects of relative timing of sex and epidemic onset only; and (*c*) SEM3 testing the effects of total sex only. Differences between the models are highlighted in the grey ovals. Arrows represent directional relationships among variables. For the best-fitting model (SEM2) positive relationships are shown with black arrows, negative ones with red arrows and partially transparent arrows denote non-significant relationships. Letters indicate the associated relationships between variables shown in figure 4, with the coefficients in electronic supplementary material, table S2.

We fitted alternative SEMs to test between three credible scenarios: SEM1 proposed that both host total population investment in sex and relative timing of host investment in sex and epidemic progression explained variation in future host diversity; SEM2 proposed that only the relative timing of host investment in sex and epidemic progression explained variation in future host diversity; and SEM3 proposed that only host total population investment in sex explained variation in future host diversity. To find our best model (and thus most plausible scenario) we evaluated the Akaike information criterion, corrected for small sample size (AICc). Before fitting our SEM, we centered (subtracting the mean) and scaled (dividing by one standard deviation) each of our explanatory variables; we also visually inspected diagnostic plots for each component of the piecewise model to ensure the assumptions were met. We started by fitting a saturated model including all causal relationships from the mixing treatment through the variables representing total investment in sex, the relative timing and investment in sex, the consequences on host diversity, and finally future epidemic size (figure 2*a*). Of these paths, the link between the parameters representing relative timing and total investment in sex was fit as a covariance for two reasons: we did not predict (nor can we envision) a causal relationship, but the parameters are coefficients from nonlinear equations that tend to correlate. Models without this covariance term returned marginally significant tests of independence, suggesting the lack of an important path between these two quantities. We then conducted Fisher's *C* tests (Shipley's tests of directed separation; see [50] to discover potentially relevant relationships that had been excluded from the model.

## 3. Results

**Figure 3.**
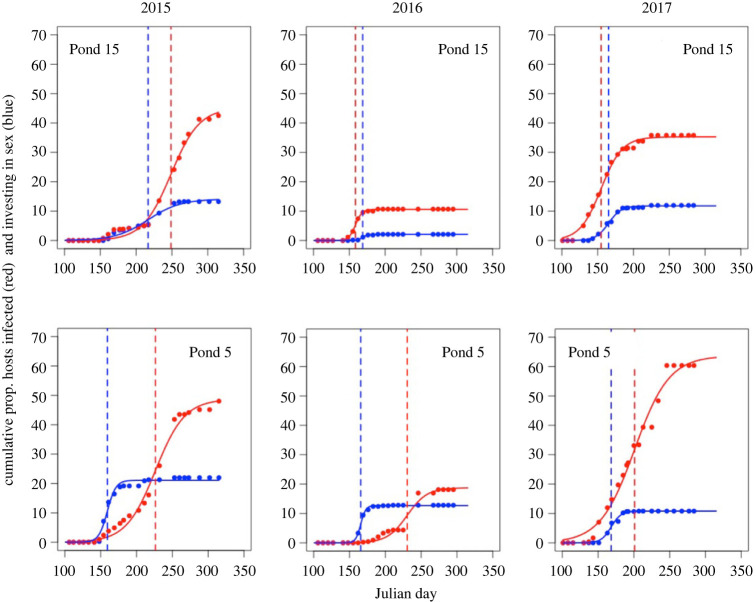
Cumulative proportion of hosts infected with *Pasteuria ramosa* (red) and carrying sexually produced dormant eggs (blue) in two example populations (of 20) across the 2015, 2016 and 2017 growing seasons. These demonstrate variation in both the timing and magnitude of both epidemics and host population investment in sex. Solid lines are the logistic model fits and dashed vertical lines denote the timing of the mid-point of the epidemic (red) and host population investment in sex (blue).

Each population was initiated with the same suite of host genotypes and parasite isolates, yet there was remarkable natural variation in the overall size, timing and synchrony of both host investment in sex and epidemic progression across populations and across years (figure 3). Late epidemics tended to be larger in 2015 and 2017 and were of longer duration in years 2016 and 2017 (i.e. had a greater scale coefficient; electronic supplementary material, table S1). Further, host populations that invested in sexual reproduction relatively late did so over a more extended period of time (again, evidenced by a greater scale coefficient) in 2015 and 2016, but not in 2017 (electronic supplementary material, table S2).

We found that host population genetic diversity (H_exp_) varied according to year, but not physical flux treatment (linear mixed effects model, year: *F*_2,38_ = 17.99, *p* < 0.0001; physical flux: *F*_1,18_ = 0.09, *p* = 0.76; electronic supplementary material, figure S1) and that population identity explained a substantial proportion of the variation in H_exp_ (0.77).

Our SEMs evaluated how the relative timings of epidemic progression and host population investment in diapause/sex, and total host population investment in sex, affected emergent host diversity and then epidemic size in the subsequent year (figure 2*a*). SEM1 included paths to future host diversity from both the relative timings of sex and epidemics and total host investment in sex (Fisher's *C* = 3.40, d.f. = 4, *p* *=* 0.49, AICc = 681.79); SEM2 modelled only the effects of relative timings on host diversity (Fisher's *C* = 5.72, d.f. = 6, *p* *=* 0.46, AICc = 680.41); and SEM3 modelled only the effect of total host sex on host diversity (Fisher's *C* = 10.80, d.f. = 6, *p* *=* 0.095, AICc = 684.84). SEM2 provided the best fit to the data, as evidenced by the lowest AICc and non-significant Fisher's *C* tests; this suggests that the relative timing of host epidemic progression and host investment in sex, and not total host investment in sex, provides the best explanation for patterns in future host diversity and future epidemic size. However, it is important to note that SEM1 provided only a marginally worse fit (ΔAICc = 1.38), though the qualitative results of SEM1 are identical (the additional path between total investment in sex and host genetic diversity is not significant; see electronic supplementary material, table S3).

**Figure 4.**
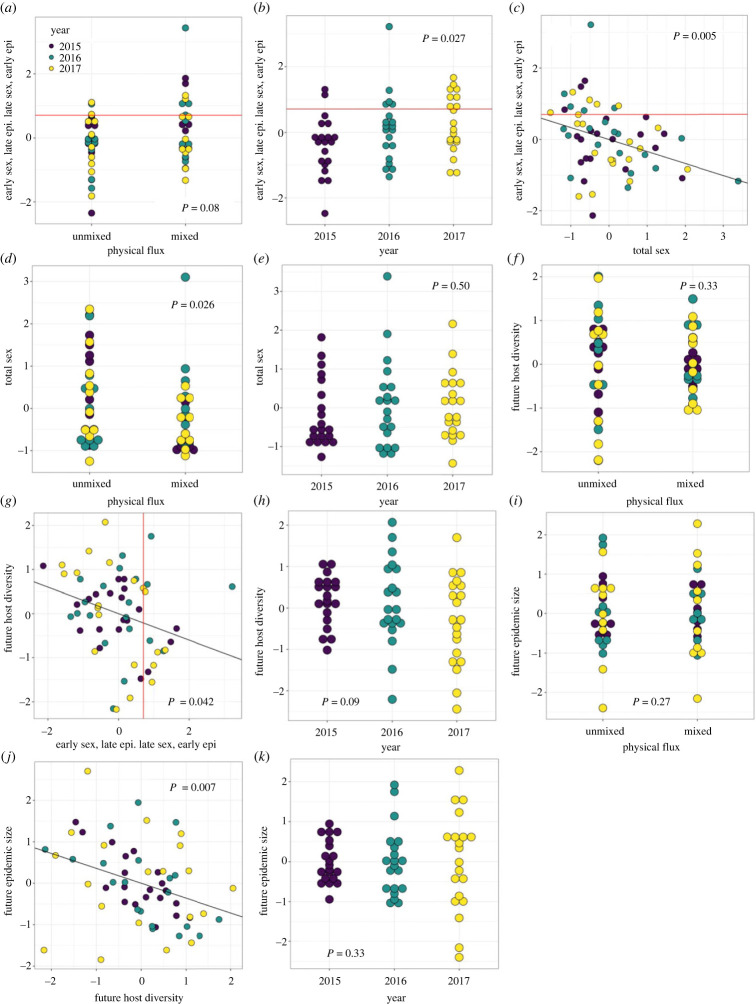
(*a–k*) Plots of residuals and partial residuals from SEM2. All variables were scaled and centred prior to analysis, and partial residuals account for all variation due to predictor variables other than the one of interest. (*a*,*b*,*c*,*g*) Simultaneous mid-epidemic and mid-host population investment in sex is shown with a red line.

Subsequent analysis of the various paths fitted by SEM2 revealed that the relative timings of host sex and epidemics were not significantly affected by physical flux treatment (table 1, figure 4*a*). The relative timings of host sex and epidemics did significantly vary across years (table 1, figure 4*b*), and was also negatively associated with total host investment in sex (i.e. when host populations invested heavily in sex, it occurred early with respect to epidemic progression; table 1, figure 4*c*). Physical flux treatment significantly affected total host investment in sex, such that sex was reduced in mixed ponds (table 1, figure 4*d*), but total host sex did not differ across years (table 1, figure 4*e*). Future host diversity (measured as H_exp_) did not differ according to physical flux (table 1, figure 4*f*), but was negatively associated with the relative timing of host sex and epidemic progression, such that diversity was highest when sex was early and epidemics were late (table 1, figure 4*g*). There was no effect of year on future host diversity (table 1, figure 4*h*). Finally, future epidemic size was unaffected by physical flux treatment (table 1, figure 4*i*), but showed a significant negative relationship with host diversity (table 1, figure 4*j*); future epidemic size did not differ according to year (table 1, figure 4*k*). As we predicted, we, therefore, found evidence linking the timing of epidemics and sex in one year and the magnitude of epidemics in the next that were mediated by the effects of epidemics on host genetic diversity.

**Table 1.** Results from SEM2 examining how the relative timings host investment in sex and epidemic progression affect host genetic diversity and disease in the future. Coeff. is the standardized model coefficient, s.e. is the standard error for the model coefficient and *p* denotes the associated *p*-value.

model	response	predictor	Coeff.	s.e.	*p*
1	rel. time sex epi.	physical flux	0.219	0.124	0.08
1	rel. time sex epi.	year	0.281	0.124	**0.0272**
2	rel. time sex epi.	total sex	−0.337	—	**0.0045**
3	total sex	physical flux	−0.288	0.126	**0.0263**
3	total sex	year	0.085	0.126	0.50
4	future host div.	physical flux	0.124	0.126	0.33
4	future host div.	rel. time sex epi.	−0.274	0.131	**0.0420**
4	future host div.	year	−0.222	0.128	0.08
5	future epi. size	physical flux	−0.359	0.127	0.27
5	future epi. size	future host div.	−0.359	0.127	**0.0065**
5	future epi. size	year	0.124	0.127	0.33

## 4. Discussion

In this study, we sought to dissect how a common and widespread host life-history strategy –the sexual production of diapausing stages—affected host genetic diversity and disease outbreak severity across multiple epidemic seasons. We did this using twenty semi-natural *Daphnia–Pasteuria* host–parasite populations that were seeded with an identical suite of host genotypes and mixed starting parasite population, where half of these populations experienced an environmental disturbance treatment (which was designed to cause variation in disease transmission dynamics [36]). We found evidence for population variation in the timings of both host investment in sex (i.e. the production of dormant offspring across space and time), with consequences for future host genetic diversity and strong effects on future epidemic size. Indeed, our structural equation modelling approach allowed us to demonstrate that host genetic diversity is a lynchpin that links past and ecological interactions with future epidemic size.

One might expect early epidemics and strong parasite-mediated selection to create a more parasite-resistant population (as seen in animal–parasite and plant–fungus systems [2,51]), and thus reduce the size of future epidemics. We find no support for this prediction, and instead show that host population genetic diversity is much more likely to act as a constraint on future epidemics, such that populations where emerging dormant stages had relatively high genetic diversity tended to suffer the smallest epidemics. We propose two non-exclusive explanations for this. First, parasite transmission is less likely to occur among hosts that are genetically dissimilar [14–16]. Second, the matching allele nature of *Daphnia–Pasteuria* infection genetics [52] means rapid *Pasteuria* adaptation to the non-dormant host population comes at the cost of maladaptation to future host populations [4], including the sexually produced daughter generation [24]. Indeed, previous work on a wild population shows that parasite evolution over the course of the epidemic means that host genotypes that were relatively resistant to parasites from one year are more susceptible to parasites from the next year and *vice versa* [24]. As a consequence, early *Pasteuria* epidemics and sex between *Daphnia* that are resistant to the current parasite population may well produce dormant offspring that are more susceptible to next year's evolved *Pasteuria* population.

Environmental disturbance caused by physical flux is known to cause smaller epidemics [4,36] and reduced host population densities [36] in this system, and we hypothesized that it could also cause host populations to invest in diapause earlier. Indeed, temperature shocks elicit diapause in the silkworm, *Bombyx mori* [53] and *Daphnia* species that inhabit temporary pools invest in diapause earlier than those native to permanent water bodies [54]. However, the relationship between physical flux and the relative timings of host population investment in temporal diapause (sex) and epidemic progression is both weak and noisy, suggesting that diapause is not an emergent adaptive strategy to this type of environmental disturbance (at least not in *Daphnia magna*).

As would be expected for complex mesocosm ecosystems, relative timing of host sex and epidemic progression did vary across years. Host populations that suffered early epidemics and invested late in sexual reproduction in a particular year were, however, much more likely to have lower genetic diversity in the following year. This fits with previous work demonstrating that over the course of the first year, host genotypic diversity declined significantly [36] as a direct consequence of parasite-mediated selection on the host populations [4], and is consistent with early epidemics stripping out genetic variation from host populations before many of the females had the opportunity to undertake the complex process of sexual reproduction (i.e. produce males, mate and release the diapausing eggs that make up the future host population; see [28]).

However, when temporal dispersal occurs before the epidemic becomes established, those diapausing offspring are shielded from selection that affects their mothers, including parasite-mediated selection, until they hatch in the future [18–20,24]. When hosts undergo temporal diapause, parasite-mediated selection on hosts is curtailed, whereas host-mediated selection on parasites continues unabated as the mother hosts return to asexual reproduction; this can reduce the impact of any disease outbreak on epidemics in future years. Importantly, we found that the overall host population investment in sex in a particular year did not predict host genetic diversity in the subsequent year: the SEM that included the path between total host sex and future epidemic size did not fit as well as the model that excluded this path. In any case, the included path between total sex and future epidemic size was not significantly different from zero (electronic supplementary material, table S3). This supports the idea that it is the timing of host temporal dispersal that is important and not total genetic recombination.

There has been considerable research into how parasite-mediated selection can affect host genetic diversity, and how host genetic diversity can affect disease outbreak size [13] over the course of individual epidemics. Our study goes a step further by demonstrating how host genetic diversity and disease epidemics can influence each other across multiple seasons in a host–parasite system where the host produces diapausing stages: a ubiquitous host life-history strategy that is poorly understood in the context of disease.

## Data Availability

Data are available on Dryad [55]. The data are provided in electronic supplementary material [56].
